# *Curcuma longa* Hepatotoxicity: A Baseless Accusation. Cases Assessed for Causality Using RUCAM Method

**DOI:** 10.3389/fphar.2021.780330

**Published:** 2021-10-29

**Authors:** Gianmarco Stati, Francesco Rossi, Silvia Sancilio, Mariangela Basile, Roberta Di Pietro

**Affiliations:** ^1^ Department of Medicine and Ageing Sciences, G. d’Annunzio University of Chieti-Pescara, Chieti, Italy; ^2^ Department of Molecular Sciences and Nanosystems, Ca’ Foscari University, Venice, Italy; ^3^ Biophysics Group, Department of Physics and Astronomy, University College London, London, United Kingdom

**Keywords:** *Curcuma longa*, curcumin, piperine, food supplement, hepatotoxicity, bioavailability enhancer, RUCAM

## Abstract

*Curcuma longa* is a perennial herb that belongs to the Zingiberaceae family. To date, literature includes more than 11.000 scientific articles describing all its beneficial properties. In the last 3 decades various surveys by the U.S. Food and Drug Administration (FDA) concluded that curcumin, the most active ingredient of the drug, is a “generally safe” compound with strong anti-oxidant effects. *Curcuma longa* was introduced in the daily diet by ayurvedic teachers due to its beneficial effects on health. Nonetheless, recently several reports, from the various global surveillance systems on the safety of plant products, pointed out cases of hepatotoxicity linked to consumption of food supplements containing powdered extract and preparations of *Curcuma longa*. The latest trend is the use of *Curcuma longa* as a weight-loss product in combination with piperine, which is used to increase its very low systemic bioavailability. Indeed, only 20 mg piperine, one of the alkaloids found in black pepper (*Piper nigrum*), assumed at the same time with 2 g curcumin increased 20-fold serum curcumin bioavailability. This combination of natural products is now present in several weight loss supplements containing *Curcuma longa*. The enhanced drug bioavailability caused by piperine is due to its potent inhibition of drug metabolism, being able to inhibit human P-glycoprotein and CYP3A4, while it interferes with UDP-glucose dehydrogenase and glucuronidation activities in liver. While only few cases of hepatotoxicity, assessed using Roussel Uclaf Causality Assessment Method (RUCAM) method, from prolonged intake of piperine and curcumin have been reported, it would be reasonable to speculate that the suspected toxicity of *Curcuma longa* could be due to the concomitant presence of piperine itself. Hence, not only there is the need of more basic research to understand the etiopathology of curcumin-related hepatotoxicity and of the combination curcumin-piperine, but human trials will be necessary to settle this dispute.

## Introduction

The aim of this short review is to trigger a more critical evaluation of scientific evidence existing in literature on potential hepatotoxicity of *Curcuma longa*. The revision of sources would be against the latest trend that blames this famous spice widely used for centuries. *Curcuma longa* has been used throughout human history for various purposes due to its wide range of biological activity (Sharifi-Rad et al., 2020). Curcumin was found to be the primary active component of the extract from the rhizome, known as turmeric. Curcumin is the ingredient responsible for the effects of turmeric as a drug in its long history of use in traditional Asian medicine for a wide variety of disorders.

The Compendium of Sushruta, the foundational text of Ayurveda dating to 250 BCE (Joshi et al., 2017), recommends an ointment containing turmeric, *Curcuma longa* powdered, to relieve the effects of poisoned food. It is not surprising, therefore, that curcumin is currently sold as a dietary supplement and that numerous clinical trials are ongoing to evaluate curcumin activity. In the last decade a large number of reports have been published on the beneficial effects of curcumin (Barchitta et al., 2019) and it has been repeatedly claimed that this natural product is efficient and safe for the prevention and treatment of several diseases (Abd El-Hack et al., 2021). Moreover, curcumin has been widely studied for its antioxidant, anti-inflammatory, and wound-healing effects (Menon and Sudheer, 2007; Shirban et al., 2021). This natural polyphenol is considered by some authors as a “wonder drug of life” (Gera et al., 2017) and it is categorized as a “generally recognized as safe” (GRAS) material, with a stable metabolism and low toxicity (Nelson et al., 2017).

Over recent years, food supplements containing *Curcuma longa* have been widely used by an increasing number of consumers and there is accumulating evidence that curcumin may not be so effective and safe. A number of reports have been issued that described the cases of highly probable drug-induced autoimmune hepatitis (DIAIH) ascribed to ingestion of *Curcuma longa* dietary supplement (Philips et al., 2020). That is, in contrast with the use, since ancient times, of *Curcuma longa*, as hepatoprotective (Rahmani et al., 2016; Tung et al., 2017; Peng et al., 2018) and for the treatment of digestive tract problems (Gera et al., 2017). Furthermore, in literature it is reported that curcumin may prevent oxidative stress-related liver disorder causing a series of metabolic reactions as i) decreasing the levels of alanine transaminase (ALT), aspartase transaminase (AST), and alkaline phosphatase (ALP). ii) It increases the expression of glutathione-S-transferase (GST), glutathione reductase (GR), glutathione peroxidase (GPx), superoxide dismutase (SOD) and catalase (CAT) while further iii) reducing NO production and inhibiting ROS formation (Farzaei et al., 2018).

The most common substance associated with *Curcuma longa* in its use as food supplement is piperine from *Piper nigrum L*. Black pepper (*Piper nigrum L.*) is the most used specie of pepper and it has found a worldwide use as a spice. Its history of use in traditional medicine is thousands of years old, being mentioned in Ayurvedic medicine treaties and in traditional Chinese medicine as early as the 250 BCE (Stojanović-Radić et al., 2019). The peculiar flavour of the *piper* family fruits, and its main pharmacological activity is given by piperine, a molecule of the alkaloid family. Between all the varieties of Piperaceae, *Piper Nigrum L.* contains the largest amount of piperine with certain cultivar reaching the 9% in weight of piperine content (Gorgani et al., 2017). Piperine structure consists of a benzodioxol ring connected to a chain with conjugated double bonds, which on the other extremity is attached to a piperidine moiety throughout a carbonylamide bond (Koul et al., 2000). The presence of a benzodioxol moiety in proximity with a conjugated system is a common feature of many bioactive molecules as psychoactive drugs (Almalki et al., 2020) and molecules able to inhibit the cytochrome P450 (Koul et al., 2000).

Piperine in particular plays a large role in traditional medicine for the treatment of flu-like symptoms and to increase appetite (Stojanović-Radić et al., 2019). In the last decade, a number of works have tested the medical properties of piperine as antioxidant (Vijayakumar et al., 2004), its effect on lipid metabolism (Du et al., 2020) and its anticancer capability (de Almeida et al., 2020). While the most important property of piperine, which is gaining the attention of the scientific community, is its ability to increase the bioavailability of drugs and other active molecules extracted by plants (Gorgani et al., 2017; Lee et al., 2018; Stojanović-Radić et al., 2019).

Piperine, as a component of black pepper, has been demonstrated to have a limited or no toxicity when assumed as a spice (≈ 1 pg/kg per day). The European Food Safety Authority identified 5 mg/kg per day as the no observed adverse effects level (NOAEL) (Bolognesi et al., 2015; Burdock, 2016). On the metabolic level, piperine has been shown to have fundamental effects on p-glycoprotein and many enzyme systems, leading to enhancement of the absorption and bioavailability of several therapeutic drugs. Piperine’s bioavailability enhancing property is also partly attributed to increased absorption as a result of its effect on the ultrastructure of intestinal brush border (Meghwal and Goswami, 2013).

## Influence of Piperine on Curcumin Bioavailability

Curcumin is a lipophilic polyphenol that is, nearly insoluble in water, with an oral bioavailability lower than 2%. The molecule of curcumin, 1,7-bis(4-hydroxy-3- methoxyphenyl)-1,6-heptadiene-3,5-dione (diferuloylmethane), when orally administrated, is quite stable in the acidic pH of the stomach (Wang et al., 1997), but it undergoes extensive first pass metabolism, is conjugated in the liver, and excreted in the feces. The main metabolites of this process are identified in curcumin glucuronide and curcumin sulfate (Ireson et al., 2001).

Its bioavailability can be enhanced by increasing its absorption and decreasing its metabolic clearance. The co-administration of curcumin with naturally occurring UDP-glucuronyl transferase (UGT) inhibitors, like piperine, improves curcumin bioavailability compared to curcumin alone (Shoba et al., 2007). Curcumin given alone at 2 g/kg achieves a very low serum concentration over a period of 4 h while the co-administration of curcumin in the presence of 20 mg/kg of piperine increases its serum concentration for a short period of 1–2 h post drug intake (Chen et al., 2017). Piperine strongly influences a number of enzymatic bio-transforming reactions in hepatic tissue*,* both *in vitro* and *in vivo* (Atal et al., 1985), acting as a non-specific inhibitor of drug metabolism which does not discriminate among the different forms of the cytochrome P_450_ (Dalvi and Dalvi, 1991).

**FIGURE 1:**
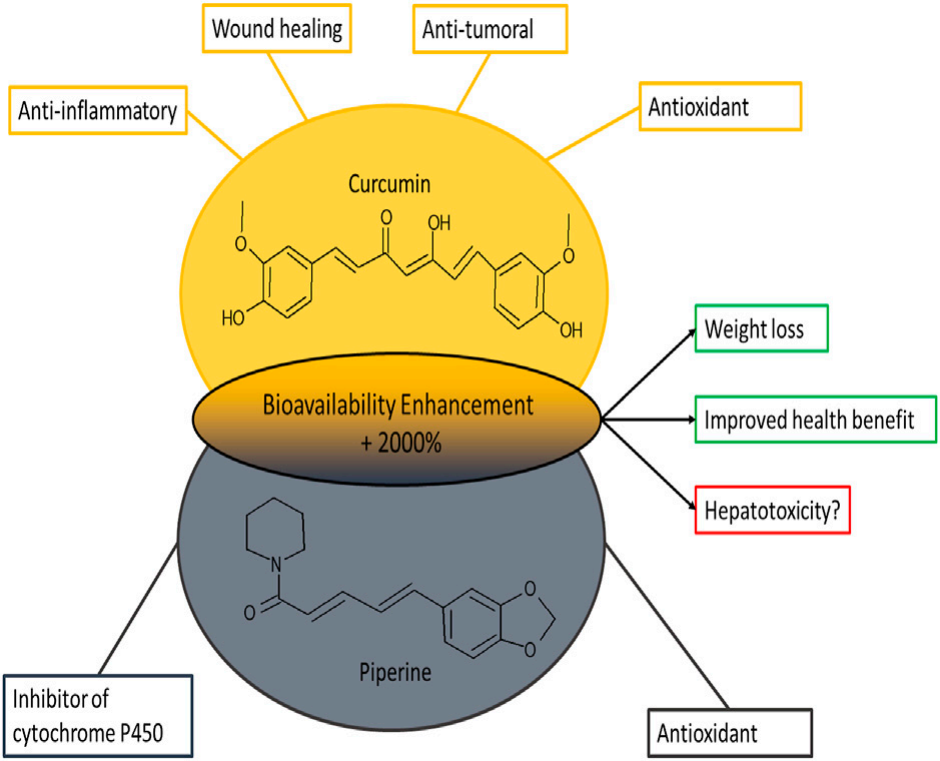
Representation of the chemical structures of curcumin and piperine and their main biological effects.

In particular, piperine strongly inhibits hepatic microsomal aryl hydrocarbon hydroxylase (AHH) and UGT (Atal et al., 1985), leading to a significantly decreased clearance of the co-administered substance and a plasma half-life significantly increased. The overall increase in the bioavailability of curcumin by piperine is of the 2000% (Shoba et al., 2007) (Figure 1).

Consuming turmeric and piperine separately is overall safe and it is even associated to a protective effect on cardiovascular mortality (Hashemian et al., 2019).

## Discussion

In recent years, numerous risk warnings related to products of natural origin have emerged. In particular, a series of reports on the possible hepatotoxicity of *Curcuma longa*, connected with the use of dietary supplements, have been published (Crijns et al., 2002; Costa et al., 2018; Lukefahr et al., 2018; Imam et al., 2019; Luber et al., 2019; Abdallah et al., 2020; Lee et al., 2020; Suhail et al., 2020; Lombardi et al., 2021). In the present article we have considered only reports who have applied Roussel Uclaf Causality Assessment Method (RUCAM) for causality assessment (Benichou et al., 1993; Danan and Benichou, 1993; Danan and Teschke, 2016), since the weaknesses of LiverTox database approach include a case selection merely based on published case number and not on a strong causality assessment method such as RUCAM (Teschke and Danan, 2021). It is nowadays the suitable method applied in 81,856 DILI and HILI cases worldwide, according to (Teschke and Danan, 2020). The considered cases were judged as probable, supporting the causal relationship to the use of *Curcuma longa* containing supplements and herb-induced liver injury (HILI).

These reports have already caused a level of response from the regulatory bodies, for example, the European Medicine Agency (EMA) has revised its guideline in the September 25, 2018 excluding the use of *Curcuma longa* supplements in case of obstruction of the bile duct, cholangitis, liver disease, gallstones, and any other biliary diseases (European Medicines Agency, 2018). A change of the policy was a consequence of this report requiring from the January 1, 2020 to add a specific warning on the label of food supplements containing curcumin, aimed at discouraging their use by people with liver disease, alterations in biliary function or with gallstones and, in case of concomitant drug intake, aimed at inviting them to consult the doctor (Ministero della Salute, 2019).

## Conclusion

The overall limited number of cases worldwide, and the few toxicity studies available together with the preliminary determinations of regulatory organs seems to exclude the possibility of an intrinsic toxicity of curcumin. At the same time no solid evidence exists that the combination of curcumin and piperine could be the cause of hepatotoxicity. This seems to exonerate the spice which for millennia has been considered a panacea for all illnesses, indicating that the supposed *Curcuma longa*-related hepatotoxicity would be a baseless accusation. Overall, these type of food supplements should get the same level of attention from regulatory organs that is, given to drugs. Ideally, all the toxicity cases should be evaluated using a comparable method, as the updated RUCAM, and made available to the scientific community. For the specific case of curcumin, the number of cases reported is still too limited for definitive answer and only a more extensive clinical trial in presence of bioavailability enhancers could definitively settle this dispute.
